# Non-invasively identifying candidates of active surveillance for prostate cancer using magnetic resonance imaging radiomics

**DOI:** 10.1186/s42492-024-00167-6

**Published:** 2024-07-05

**Authors:** Yuwei Liu, Litao Zhao, Jie Bao, Jian Hou, Zhaozhao Jing, Songlu Liu, Xuanhao Li, Zibing Cao, Boyu Yang, Junkang Shen, Ji Zhang, Libiao Ji, Zhen Kang, Chunhong Hu, Liang Wang, Jiangang Liu

**Affiliations:** 1 Department of Radiology, Beijing Friendship Hospital, Capital Medical University, Beijing, 100050 China; 2 School of Engineering Medicine, Beihang University, Beijing, 100191 China; 3 Key Laboratory of Big Data-Based Precision Medicine (Beihang University), Ministry of Industry and Information Technology of the People’s Republic of China, Beijing, 100191 China; 4 School of Biological Science and Medical Engineering, Beihang University, Beijing, 100191 China; 5 Department of Radiology, the First Affiliated Hospital of Soochow University, Suzhou, 215006 Jiangsu Province China; 6 Department of CT-MR Center, the People’s Hospital of Jimo, Qingdao, 266200 Shandong Province China; 7 Department of Radiology, Sinopharm Tongmei General Hospital, Datong, 037003 Shanxi Province China; 8 Department of Urology, Beijing Friendship Hospital, Capital Medical University, Beijing, 100050 China; 9 Department of Radiology, the Second Affiliated Hospital of Soochow University, Suzhou, 215004 Jiangsu Province China; 10 Department of Radiology, the People’s Hospital of Taizhou, Taizhou, 225399 Jiangsu Province China; 11 Department of Radiology, Changshu No. 1 People’s Hospital, Changshu, 215501 Jiangsu Province China; 12 Department of Radiology, Tongji Hospital, Tongji Medical College, Huazhong University of Science and Technology, Wuhan, 430030 Hubei Province China; 13 Beijing Engineering Research Center of Cardiovascular Wisdom Diagnosis and Treatment, Beijing, 100191 China

**Keywords:** Active surveillance, Prostate cancer, Magnetic resonance imaging, Radiomics

## Abstract

Active surveillance (AS) is the primary strategy for managing patients with low or favorable-intermediate risk prostate cancer (PCa). Identifying patients who may benefit from AS relies on unpleasant prostate biopsies, which entail the risk of bleeding and infection. In the current study, we aimed to develop a radiomics model based on prostate magnetic resonance images to identify AS candidates non-invasively. A total of 956 PCa patients with complete biopsy reports from six hospitals were included in the current multicenter retrospective study. The National Comprehensive Cancer Network (NCCN) guidelines were used as reference standards to determine the AS candidacy. To discriminate between AS and non-AS candidates, five radiomics models (i.e., eXtreme Gradient Boosting (XGBoost) AS classifier (XGB-AS), logistic regression (LR) AS classifier, random forest (RF) AS classifier, adaptive boosting (AdaBoost) AS classifier, and decision tree (DT) AS classifier) were developed and externally validated using a three-fold cross-center validation based on five classifiers: XGBoost, LR, RF, AdaBoost, and DT. Area under the receiver operating characteristic curve (AUC), accuracy (ACC), sensitivity (SEN), and specificity (SPE) were calculated to evaluate the performance of these models. XGB-AS exhibited an average of AUC of 0.803, ACC of 0.693, SEN of 0.668, and SPE of 0.841, showing a better comprehensive performance than those of the other included radiomic models. Additionally, the XGB-AS model also presented a promising performance for identifying AS candidates from the intermediate-risk cases and the ambiguous cases with diagnostic discordance between the NCCN guidelines and the Prostate Imaging-Reporting and Data System assessment. These results suggest that the XGB-AS model has the potential to help identify patients who are suitable for AS and allow non-invasive monitoring of patients on AS, thereby reducing the number of annual biopsies and the associated risks of bleeding and infection.

**Supplementary Information:**

The online version contains supplementary material available at 10.1186/s42492-024-00167-6.

## Introduction

Early detection and treatment can effectively reduce prostate cancer (PCa) mortality [1]. However, for some patients diagnosed early, PCa may not pose an immediate threat to health throughout their lifetime. Thus, immediate treatment may not benefit these patients but may result in side effects (i.e., sexual dysfunction, urinary dysfunction, and fatigue) that diminish the quality of life [2, 3].

Active surveillance (AS) refers to regular monitoring of PCa progression, during which curative treatment is administered once PCa evolves into a high-risk tumor [4]. The primary aim of AS is to delay or avoid unnecessary treatment and its corresponding undesirable effects [5]. Therefore, AS has become the primary strategy for managing patients with low- or favorable intermediate-risk (FIR) PCa [6]. According to AS protocols [7, 8], an annual biopsy is required to determine whether patients on AS require reclassification to a higher-risk category. However, repeated biopsies increase pain and the risk of infection [9, 10] and may complicate the execution of radical prostatectomy (RP) [11].

Magnetic resonance imaging (MRI) is a non-invasive imaging method that can provide high spatial resolution and overall morphological characterization of tumors [12, 13]. In particular, the standardized assessment method, known as the Prostate Imaging-Reporting and Data System v.2 (PI-RADSv2), has been reported to be crucial in identifying suitable AS candidates [14, 15]. However, the PI-RADSv2 assessment relies on a semi-quantitative interpretation of MRI images and greatly depends on the radiologist’s experience, resulting in substantial variability in the assessment results among different radiologists [15–17]. Additionally, the visual assessment by radiologists may overlook some of the non-visible information from the tumors.

Gaur [18] suggested the use of radiomics in AS for PCa. Radiomics methods can extract high-throughput features, even those not visible to the naked eye from medical images that may reflect tumor phenotypes [19–21] and output a quantitative score indicating the risk probability of the tumor [22]. Recent studies have discovered that radiomics methods could predict the progression of AS in patients [23, 24]. For instance, Algohary et al. [23] developed a radiomic model to identify clinically significant PCa in patients undergoing AS. Sushentsev et al. [24] developed a radiomic model to predict the histopathological progression of PCa in patients undergoing AS. However, none of these studies identified suitable AS candidates due to limited sample sizes and the absence of independent external validation [23, 24]. Therefore, the current study aimed to develop and externally validate a radiomics model using a multicenter dataset to non-invasively discriminate patients with PCa who qualify for AS from those who should undergo definitive treatments, such as RP.

## Methods

### Patients and MRI techniques

**Fig. 1:**
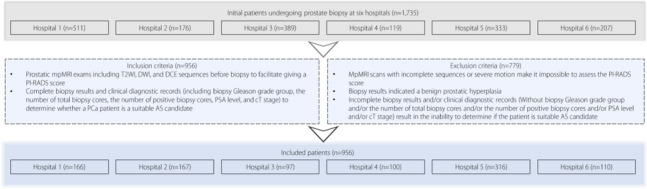
Overview of patients based on the inclusion and exclusion criteria and allocation of patients in the training and external validation cohorts. cT stage: clinical tumor stage; DCE: Dynamic contrast-enhanced; DWI: Diffusion-weighted imaging; mpMRI: Multiparametric magnetic resonance imaging; PSA: Prostate-specific antigen; T2WI: T2-weighted imaging

The local Institutional Ethics Review Board approved the study and waived the requirement for written informed consent owing to its retrospective nature. This study adhered to the 1964 Declaration of Helsinki and its subsequent guidelines. Overall, 1,735 consecutive patients who underwent prostate biopsy at six hospitals between January 2018 and June 2021 were enrolled. Based on the inclusion and exclusion criteria (Fig. 1), 956 patients (166, 167, 97, 100, 316, and 110 from hospitals 1 (H1), 2 (H2), 3 (H3), 4 (H4), 5 (H5), and 6 (H6), respectively) were included in the study.

All patients underwent 3.0-T MRI using an abdominal phased-array coil before prostate biopsy (Supplementary Table S1).

### Biopsy analysis, PI-RADS assessment, and lesion annotation

The biopsy results for H1, H2, H4, H5, and H6 were obtained using transrectal ultrasound (TRUS)-guided systemic biopsy and MRI-guided targeted biopsy, and those for H3 were obtained using TRUS-guided saturation biopsy. At each hospital, a junior pathologist analyzed the samples, and the results were verified by a senior pathologist. Disagreements were resolved through discussions between the readers.

According to PI-RADSv2.1 [15], eight junior radiologists (JR1–8) and three experienced radiologists (ER1–3) with over 3 and 18 years of experience, respectively, participated in image interpretation. After the PI-RADS assessment, the same junior radiologist delineated the prostate lesions from the T2-weighted (T2W) images. The delineated lesion was referred to as the region of interest (ROI). The PI-RADS assessment and lesion annotation details are described in Supplementary Sect. 1.

### Reference standard

According to the National Comprehensive Cancer Network (NCCN) guidelines [8], patients with PCa undergoing AS meet one of the following criteria: (1) PSA level < 10 ng/mL, cT2b–cT2c, Gleason grade group (GGG) 1, and < 50% positive biopsy cores, or (2) PSA level < 10 ng/mL, cT1–cT2a, GGG 2, and < 50% positive biopsy cores, or (3) PSA level 10–20 ng/mL, cT1–cT2a, GGG 1, and < 50% positive biopsy cores, or (4) PSA level < 10 ng/mL, cT1–cT2a, GGG 1, and < 50% positive biopsy cores. Additionally, the patients with GGG ≥ 3 and ≤ 2 were classified as having clinically significant PCa (csPCa) and non-csPCa, respectively [25].

### Development and validation of the radiomics model

**Fig. 2:**
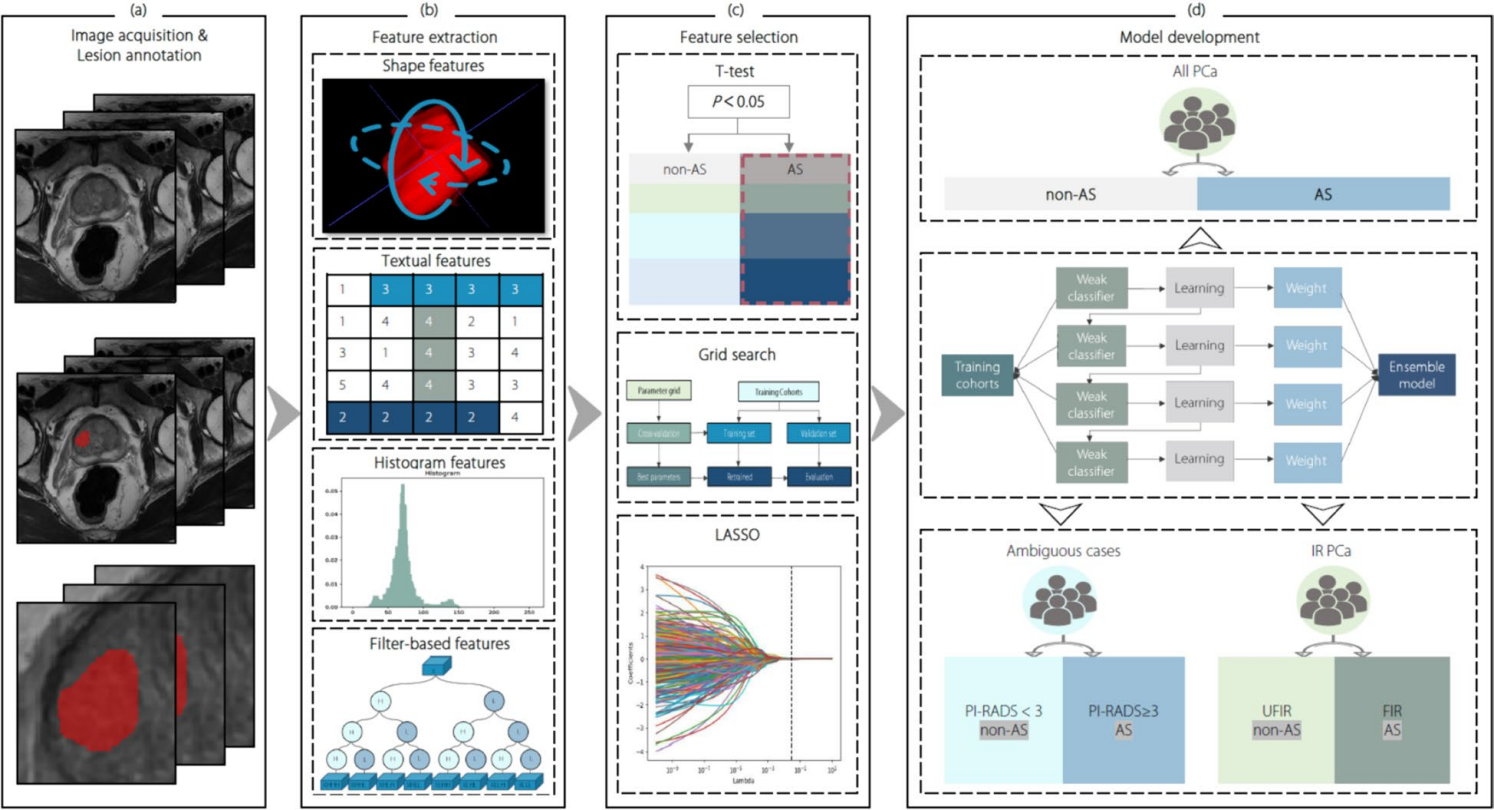
AS candidate classification radiomics model workflow pipeline. **a** MR images were exported through the post-processing workstation. For the lesions on T2W images, the ROI were manually annotated slice by slice; (**b**) The radiomics features, including shape, texture, histogram, and filter-based features, were extracted; (**c**) Using a t-test, highly differentiated features were selected to distinguish AS from non-AS candidates. Then, LASSO with a five-fold cross-validation was implemented for further feature selection; (**d**) Using the features selected by LASSO, a radiomics model was constructed based on the traditional machine learning model (e.g., the XGBoost classifier). Two subgroup analyses were performed to further evaluate this radiomics model’s performance, including distinguishing AS from the ambiguous case group and the immediate-risk group. IR PCa: Immediate-risk prostate cancer; LASSO: Least absolute shrinkage and selection operator; MR: Magnetic resonance

Figure 2 illustrates the workflow pipeline of constructing a radiomics models (e.g., eXtreme Gradient Boosting (XGBoost)). Considering the easy acquisition and abundant texture information, T2W images were used to construct the radiomics model [26, 27]. First, images were preprocessed (Supplementary Sect. 2). Next, for each participant, 1,595 radiomics features were extracted from the ROI of the original T2W and the derived images (Supplementary Sect. 3). Then, after feature selection, the radiomics features that were most correlated with the classification were selected from the 1,595 radiomics features (Supplementary Sect. 4). Additionally, XGBoost, logistic regression (LR), random forest (RF), adaptive boosting (AdaBoost), and decision tree (DT) classifiers were used to develop classification models based on the selected radiomic features to identify AS candidates. These radiomics models were referred to as XGBoost AS classifier (XGB-AS), LR AS classifier (LR-AS), RF AS classifier (RF-AS), AdaBoost AS classifier (AdaB-AS), and DT AS classifier (DT-AS), respectively.

A three-fold cross-center validation was conducted for each model (i.e., LR-AS, RF-AS, AdaB-AS, DT-AS, XGB-AS), with four hospitals used as a training cohort (TC) and the remaining two hospitals used as an external validation cohort (EVC) for each fold of cross-validation, ensuring that the models were multi-center trained and multi-center tested. The details in the data splitting for each fold of the three-fold cross-center were summarized in Supplementary Table S2. Specifically, for the first fold (Fold 1), patients from H1-4 (*n* = 530) and those from H5-6 (*n* = 426) were divided into TC and EVC; for the second fold (Fold 2), patients from H1, 2, 5, and 6 (*n* = 759) and those from H3-4 (*n* = 197) were divided into TC and EVC; for the third fold (Fold 3), patients from H3-6 (*n* = 623) and those of H1-2 (*n* = 333) were divided into TC and EVC.

In each fold of the three-fold cross-center validation, the models were developed using open-source packages in Python (v.3.7), including Scikit-learn and xgboost (v.1.6.2). The hyperparameters for these models were optimized using GridSearch CV. GridSearchCV is a package within the Scikit-learn library that consists of two main elements: grid search, which is used to enumerate the hyperparameters and search for the optimal ones, and cross-validation (five-fold cross-validation for the current study), which is used to assess the model’s performance across different subsets of TC.

Owing to the imbalance between the number of AS and non-AS cases, the classification threshold was determined by the threshold-moving method [28], namely, $\frac{{n}_{AS}}{{n}_{AS}+{n}_{non-AS}}$, where ${n}_{AS}$ and ${n}_{non-AS}$ refer to the number of AS and non-AS cases in TC of the corresponding fold of the three-fold cross-center validation, respectively (Supplementary Table S2). Thus, if the output score of the radiomics model for a case exceeded the threshold, the case was classified into the AS group; otherwise, it was classified into the non-AS group. In agreement with clinical practice, the non-AS group (requiring immediate treatment) was designated as positive cases, and the AS group was designated as negative cases.

The means of area under the receiver operating characteristic curve (AUC), accuracy (ACC), sensitivity (SEN), and specificity (SPE) for the included radiomics models (i.e., XGB-AS, LR-AS, RF-AS, AdaB-AS, and DT-AS) across the three-fold cross-validation were calculated. AUC reflected the overall performance of the classification model without dependence on the threshold, and therefore, it was used to compare the performance of the models (i.e., XGB-AS, LR-AS, RF-AS, AdaB-AS, and DT-AS) for identifying AS candidates.

### Subgroup analysis

Two subgroup analyses were conducted in EVC, using ACC to evaluate the performance of the model, as described below.

1. Identifying AS candidates with discordance in their assessment results between the PI-RADS [15] and NCCN guidelines [8]: In clinical practice, patients with PI-RADS < 3 are not considered for biopsy due to the relatively low risk of csPCa, whereas those with PI-RADS ≥ 3 necessitate biopsy confirmation due to the relatively high risk of csPCa [15, 29, 30]. However, taking the EVC of Fold 1 as an example, 36 patients among those with PI-RADS < 3 did not qualify for AS, according to the NCCN guidelines [8]. In contrast, 42 patients among those with PI-RADS ≥ 3 (i.e., 34 patients with PI-RADS > 3 and eight patients with PI-RADS = 3) were considered suitable for AS according to the NCCN guidelines [8]. Thus, for these 78 ambiguous cases, we evaluated whether XGB-AS could aid in identifying AS candidates and therefore, reducing the unnecessary biopsies.
2. Identifying AS candidates from the intermediate-risk group: According to the NCCN guidelines [8], patients with PCa classified as intermediate-risk included those with FIR and unfavorable intermediate-risk (UFIR) (Supplementary Sect. 5). AS was considered as a treatment option for FIR patients but not for UFIR patients [8]. However, differentiating between patients with FIR and those with UFIR based on MRI images is difficult. Therefore, we investigated whether the best-performing model could identify AS candidates in the intermediate-risk group. Also taking the EVC of Fold 1 as an example, 85 patients classified as intermediate-risk (FIR, *n* = 37; UFIR, *n* = 48) were selected for this subgroup analysis.

### Statistical analyses

To assess the intergroup differences in the proportion of AS candidates between TC and EVC in the three-fold cross-center validation, the *χ*^2^ test was performed. Moreover, *P* < 0.05 indicated statistical significance. The AUC with 95%CIs was used to evaluate performance, and DeLong’s test was used to examine the difference in AUC between the radiomics model and PI-RADS assessment. RStudio (v.4.0.3), Statistical Package for Social Sciences (v.26.0 IBM, Armonk, NY, USA), and Python (v.3.7) were used for statistical analyses.

## Results

### Patient characteristics

Overall, 956 patients with PCa who underwent 3.0-T MRI at six hospitals were included. The clinical characteristics, demographic information, and distribution of AS and non-AS candidates for included patients are summarized in Table 1. According to the NCCN guidelines [8], for Fold 1, 17.2% of patients (91/530) in TC and 12.9% of patients (55/426) in EVC met the AS criteria; for Fold 2, 15.9% of patients (121/759) in TC and 12.7% of patients (25/197) in EVC met the AS criteria; for Fold 3, 12.8% of patients (80/623) in TC and 19.8% of patients (66/333) in EVC met the AS criteria (Supplementary Table S2).

**Table 1:** Descriptive characteristics and distribution of AS and non-AS candidates from six hospitals

Characteristic	H1 (*n* = 166)	H2 (*n* = 167)	H3 (*n* = 97)	H4 (*n* = 100)	H5 (*n* = 316)	H6 (*n* = 110)
**MRI strength and vendor**	3.0 T, Siemens	3.0 T, Philips	3.0 T, Siemens	3.0 T, Philips	3.0 T, Siemens, GE, Philips	3.0 T, Siemens
**Age, median (IQR) (years)**	71 (66, 75)	72 (67, 78)	72 (65, 77)	73 (66, 77)	69 (65, 76)	74 (69, 78)
**Biopsy GGG**	**166**	**167**	**97**	**100**	**316**	**110**
1	24 (14.5%)	66 (39.5%)	15 (15.5%)	7 (7.0%)	35 (11.1%)	7 (6.4%)
2	47 (28.3%)	24 (14.4%)	18 (18.6%)	35 (35.0%)	49 (15.5%)	13 (11.8%)
3	46 (27.7%)	25 (15.0%)	15 (15.5%)	19 (19.0%)	54 (17.1%)	20 (18.2%)
4	24 (14.5%)	20 (12.0%)	27 (27.8%)	29 (29.0%)	64 (20.3%)	36 (32.7%)
5	25 (15.0%)	32 (19.1%)	22 (22.6%)	10 (10.0%)	114 (36.0%)	34 (30.9%)
**Percentage of positive biopsy cores**	**166**	**165**	**97**	**100**	**313**	**96**
< 50%	96 (57.8%)	113 (68.5%)	37 (38.1%)	34 (34.0%)	193 (61.7%)	19 (19.8%)
≥ 50%	70 (42.2%)	52 (31.5%)	60 (61.9%)	66 (66.0%)	120 (38.3%)	77 (80.2%)
**cT stage**	**166**	**167**	**97**	**100**	**316**	**110**
cT1–cT2a	52 (31.3%)	50 (29.9%)	14 (14.4%)	35 (35.0%)	115 (36.4%)	21 (19.1%)
cT2b–cT2c	61 (36.7%)	77 (46.1%)	21 (21.6%)	27 (27.0%)	71 (22.5%)	54 (49.1%)
≥ cT3a	53 (32.0%)	40 (24.0%)	62 (64.0%)	38 (38.0%)	130 (41.1%)	35 (31.8%)
**PSA (ng/mL)**	**164**	**167**	**97**	**100**	**310**	**90**
0 ≤ PSA < 10	53 (32.3%)	81 (48.5%)	16 (16.5%)	23 (23.0%)	101 (32.6%)	10 (11.1%)
10 ≤ PSA < 20	53 (32.3%)	53 (31.7%)	18 (18.6%)	26 (26.0%)	70 (22.6%)	13 (14.4%)
PSA ≥ 20	58 (35.4%)	33 (19.8%)	63 (64.9%)	51 (51.0%)	139 (44.8%)	67 (74.5%)
**Label**	**166**	**167**	**97**	**100**	**316**	**110**
non-AS	155 (93.4%)	112 (67.1%)	89 (91.8%)	83 (83.0%)	263 (83.2%)	108 (98.2%)
AS	11 (6.6%)	55 (32.9%)	8 (8.2%)	17 (17.0%)	53 (16.8%)	2 (1.8%)
**PI-RADS score**	**166**	**167**	**97**	**100**	**316**	**110**
1	0 (0.0%)	1 (0.6%)	1 (1.0%)	0 (0.0%)	1 (0.3%)	1 (0.9%)
2	12 (7.2%)	17 (10.2%)	1 (1.0%)	4 (4.0%)	37 (11.7%)	10 (9.1%)
3	10 (6.0%)	29 (17.4%)	8 (8.2%)	34 (34.0%)	15 (4.7%)	14 (12.7%)
4	33 (19.9%)	74 (44.3%)	9 (9.3%)	15 (15.0%)	75 (23.7%)	13 (11.8%)
5	111 (66.9%)	46 (27.5%)	78 (80.5%)	47 (47.0%)	188 (59.6%)	72 (65.5%)

*IQR* Interquartile range

### Model validation and comparison

Table 2 summarizes the means of the AUC, ACC, SEN, and SPE of the included radiomics models (i.e., LR-AS, RF-AS, AdaB-AS, DT-AS, and XGB-AS) for identifying AS candidates across three-fold cross-center validation. As indicated in Table 2, XGB-AS has the highest AUC (0.803) and ACC (0.693). Compared to XGB-AS, DT-AS and AdaB-AS show higher SEN (0.752 *vs* 0.668) and SPE (0.865 *vs* 0.841), respectively. However, DT-AS and AdaB-AS presented much lower SPE (0.539 *vs* 0.841) and SEN (0.491 *vs* 0.668) when compared to XGB-AS, respectively. These results indicate that XGB-AS exhibits better comprehensive performance in identifying AS candidates than the other models.

**Table 2:** Mean performance of the included radiomics models for identifying AS candidates across three-fold cross-center validation

Model	AUC	ACC	SEN	SPE
XGB-AS	**0.803**	**0.693**	0.668	0.841
LR-AS	0.749	0.691	0.692	0.670
DT-AS	0.705	0.668	**0.752**	0.539
RF-AS	0.751	0.639	0.610	0.784
AdaB-AS	0.751	0.553	0.491	**0.865**

Bold numbers represent the highest values of the performance metrics in each column

*LR-AS* Radiomics model based on the Logistic Regression classifier for identifying AS candidates, *RF-AS* Radiomics model based on the Random Forest classifier for identifying AS candidates, *AdaB-AS* Radiomics model based on the Adaptive Boosting classifier for identifying AS candidates, *DT-AS* Radiomics model based on the Decision Tree classifier for identifying AS candidates, *XGB-AS* Radiomics model based on the eXtreme Gradient Boosting classifier for identifying AS candidates

For the convenience of description, the XGB-AS models trained and tested in Folds 1, 2, and 3 are referred to as XGB-AS-1, XGB-AS-2, and XGB-AS-3, respectively, whose AUC, ACC, SEN and SPE were summarized in Table 3. As indicated in Table 3, the AUC of XGB-AS-2 is slightly higher than that of XGB-AS-1, which is much higher than that of XGB-AS-3. The detailed optimal hyperparameters of XGB-AS-1, XGB-AS-2, and XGB-AS-3 were summarized in Supplementary Table S3. Additionally, as indicated in Table 3, there is no significant difference in the proportion of AS candidates between TC and EVC for Fold 1 (*P* = 0.069) or Fold 2 (*P* = 0.259) of the three-fold cross-center validation. In contrast, such difference is significant for Fold 3 of the three-fold cross-center validation (*P* = 0.0043), which may be one of the reasons for the decrease in the performance of XGB-AS-3. Thus, to minimize the bias resulting from the patients splitting during the three-fold cross-center validation, the model with the median performance according to AUC (i.e., XGB-AS-1) was selected as the most clinically applicable model, which was used for the subgroup analyses in the corresponding EVC to further validate its clinical performance.

**Table 3:** Performance of the XGB-AS model developed in each fold of threefold cross-centre validation

XGB-AS model	AS% in TC	AS% in EVC (*P*-value)	AUC	ACC	SEN	SPE
XGB-AS-1	17.3%	12.9% (*P* = 0.069)	0.851	**0.779**	**0.771**	0.836
XGB-AS-2	15.9%	12.7% (*P* = 0.259)	**0.866**	0.665	0.622	**0.960**
XGB-AS-3	12.8%	19.8% (*P* = 0.0043)	0.693	0.634	0.610	0.727

Bold numbers represent the highest values of the performance metrics in each column. The *P*-value was calculated using a *χ*^2^ test to compare the AS% between the TC and EVC for the corresponding fold of cross-center validation

*XGB-AS* radiomics model based on the eXtreme Gradient Boosting classifier for identifying AS candidates; *AS%* the proportion of active surveillance candidates; *XGB-AS-1* the XGB-AS model developed in the first fold of the three-fold cross-center validation; *XGB-AS-2* the XGB-AS model developed in the second fold of the three-fold cross-center validation; *XGB-AS-3* the XGB-AS model developed in the third fold of the three-fold cross-center validation

**Fig. 3:**
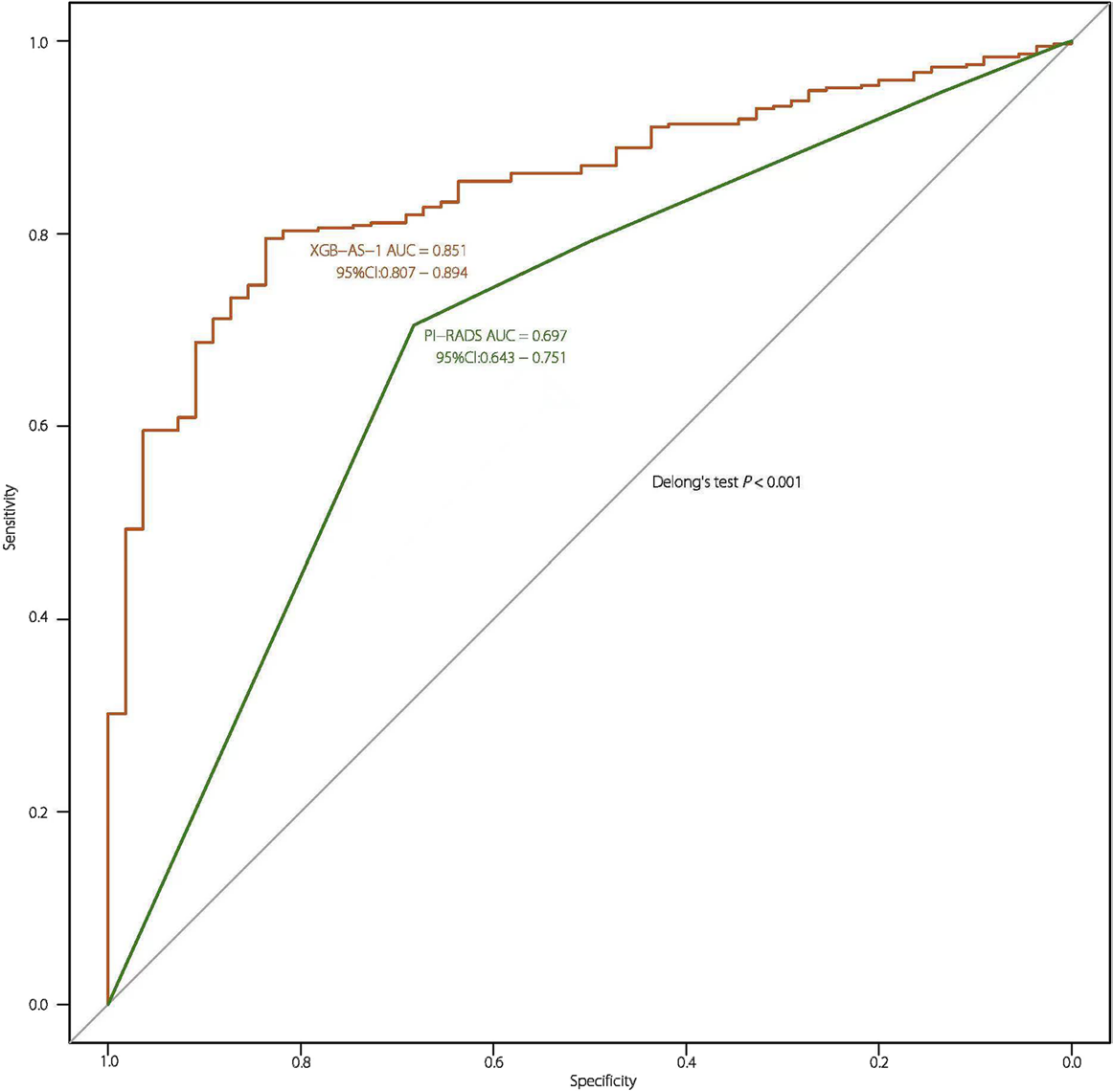
Receiver operating characteristic curves for comparisons between the XGB-AS-1 and PI-RADS performed by experienced radiologists

In clinical practice, the PI-RADS assessment is often used to determine whether a biopsy is needed to confirm the presence of csPCa [15]. This can reduce the incidence of unnecessary biopsies by improving the csPCa detection rate [25]. Similarly, XGB-AS-1 was developed to non-invasively identify AS candidates, thereby reducing the need for unnecessary biopsies. Therefore, to reduce unnecessary biopsies, we compared XGB-AS-1 with PI-RADS assessment. In the EVC, XGB-AS-1 yielded an AUC of 0.851 (95%CI: 0.807–0.894) for identifying AS candidates, which was significantly larger than that of the PI-RADS assessment (0.697, 95%CI: 0.643–0.751; *P* < 0.001; Fig. 3). The ACC, SEN, and SPE of XGB-AS-1 were 0.779 (332/426), 0.771 (286/371), and 0.836 (46/55) in the EVC, respectively. In the PI-RADS assessment, when a threshold of PI-RADS ≥ 3 was employed for detecting csPCa, the ACC, SEN, and SPE in the EVC were reported as 0.735 (313/426), 0.910 (293/322), and 0.192 (20/104), respectively [29, 30].

### Subgroup analysis

**Fig. 4:**
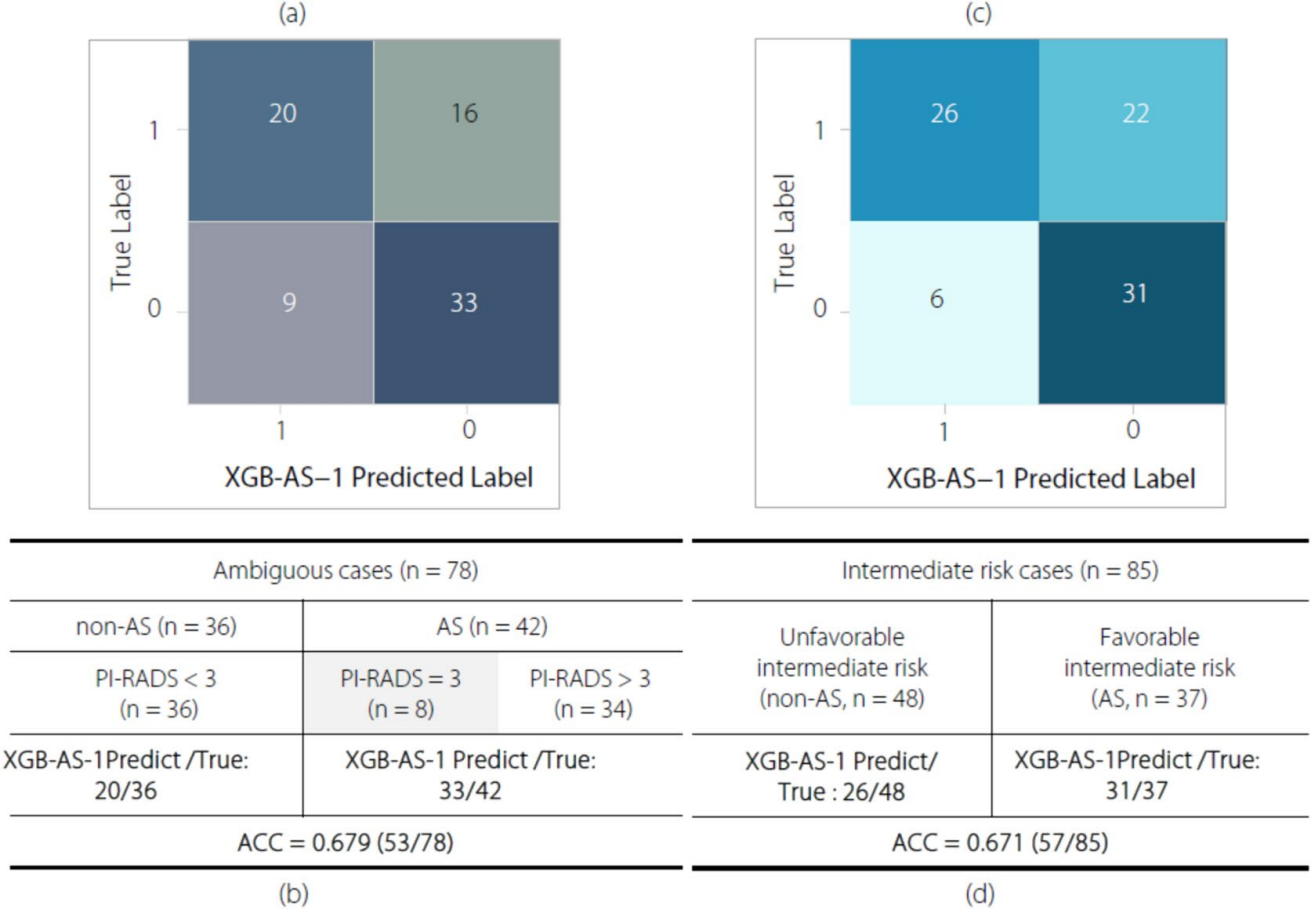
The results of subgroup analysis results for XGB-AS-1. Panels (**a**) and (**b**) show the confusion matrix and ACC for distinguishing AS from ambiguous cases with discordance in the assessment results between the PI-RADS assessment and NCCN guidelines, respectively. Panels (**c**) and (**d**) show the confusion matrix and ACC for distinguishing FIR patients (AS candidates) from intermediate-risk patients, respectively. The *True Label* and *XGB-AS-1 predicted label* indicate the ground truth and classification result of the XGB-AS-1 model, respectively, with 1 for non-AS and 0 for AS. The value in each color check indicates the number of cases for the corresponding *True Label* and *XGB-AS-1 predicted label*. *XGB-AS-1,* the most clinically applicable radiomics model developed in this study based on the XGBoost architecture for identifying AS candidates

As revealed by the first subgroup analysis (Fig. 4a), among 78 ambiguous cases characterized by the discordance between the PI-RADS assessment and NCCN guidelines, 78.6% (33/42) of the AS candidates with PI-RADS ≥ 3 and 55.6% (20/36) of the non-AS candidates with PI-RADS < 3 were accurately identified (Fig. 4b). Therefore, XGB-AS-1 had an ACC of 67.9% (53/78) in discriminating the ambiguous cases.

As revealed by the second subgroup analysis (Fig. 4c), among the 85 intermediate-risk patients, 83.8% (31/37) of AS and 54.2% (26/48) of non-AS candidates were identified from the FIR and UFIR patients, respectively (Fig. 4d). Therefore, the XGB-AS-1 model had an ACC of 67.1% (57/85) for identifying AS candidates in the intermediate-risk group.

### Feature analysis

Table 4 summarizes the features selected for the development of XGB-AS-1. As indicated in Table 4, only three categories of features (i.e., one original feature, nine wavelet features, and three local binary pattern in 3D (LBP-3D) features) were selected by the feature selection process to develop XGB-AS-1 (Supplementary Figure S1). Among these features, when comparing the AS group to the non-AS group, four features (including one original feature, two wavelet features, and one LBP-3D feature) had significantly higher values for the AS group, whereas the remaining nine features (consisting of seven wavelet features and two LBP-3D features) demonstrated significantly lower values for the AS group (*P* < 0.05).

**Table 4:** The difference in the value of features selected by the feature selection for the development of XGB-AS-1 between AS and non-AS candidates in the corresponding TC

Feature name	AS candidate (median)	Non-AS candidate (median)	*P*-value
original_shape_Sphericity	0.693	0.635	< 0.001
wavelet-HHL_glrlm_LongRunLowGrayLevelEmphasis	2.344	3.260	< 0.001
wavelet-LLH_glrlm_LongRunLowGrayLevelEmphasis	3.007	4.478	< 0.001
wavelet-LHL_firstorder_Maximum	0.623	1.113	0.016
wavelet-LHL_firstorder_Skewness	-0.005	0.078	< 0.001
wavelet-LLL_glszm_SmallAreaEmphasis	0.021	0.278	< 0.001
wavelet-LLH_glszm_ZoneEntropy	1.552	1.922	< 0.001
wavelet-HLL_glrlm_GrayLevelNonUniformityNormalized	0.501	0.500	< 0.001
wavelet-LLH_firstorder_RootMeanSquared	0.145	0.159	0.021
wavelet-HLL_glrlm_HighGrayLevelRunEmphasis	2.557	2.522	< 0.001
lbp-3D-k_firstorder_Skewness	1.267	1.424	< 0.001
lbp-3D-k_gldm_SmallDependenceHighGrayLevelEmphasis	0.078	0.055	< 0.001
lbp-3D-m2_firstorder_Maximum	18.834	19.340	< 0.001

## Discussion

In this study, a radiomics model based on MRI was developed and externally validated to discriminate between AS and non-AS candidates. The results indicated that XGB-AS demonstrated promising performance in identifying AS candidates. According to AS protocols [7, 8], patients on AS must periodically undergo repeat biopsies to determine whether they can continue to follow AS. However, frequent biopsies lead to side effects such as bleeding and infection [9, 10], and a particularly difficult implementation of RP [11]. Furthermore, XGB-AS accurately identified an average of 84.1% of AS candidates. If XGB-AS had been utilized previously, patients with PCa could have avoided unnecessary biopsies, the risk of overtreatment, and potentially challenging RP. Thus, XGB-AS can serve as a primary non-invasive categorization tool, assisting in the accurate identification of AS candidates and avoiding the detrimental effects of repeated biopsies.

In terms of identifying patients with PCa who required biopsy confirmation, XGB-AS displayed better performance than the PI-RADS assessment conducted by experienced radiologists. Moreover, disagreement exists regarding whether biopsy confirmation is required between the PI-RADS assessment [15] and NCCN guidelines [8]. The proposed XGB-AS-1 accurately identified 78.6% (33/42) of AS candidates and 55.6% (20/36) of non-AS candidates from ambiguous cases, with discordance in the assessment results between the PI-RADS assessment and the NCCN guidelines (i.e., the reference standard of the current study). Thus, when patients with PCa were assessed using MRI, our model effectively reduced unnecessary biopsies and enhanced detection SEN and SPE. Therefore, our model may be a potential tool to aid radiologists in the risk stratification of PCa based on non-invasive MRI images. According to clinical practice guidelines [8], an invasive biopsy is necessary for risk stratification of patients with FIR and UFIR. However, utilizing XGB-AS-1, 83.8% (31/37) of the FIR patients who were suitable AS candidates and 54.2% (26/48) of the UFIR patients who were non-AS candidates were correctly identified using MRI. Thus, unnecessary biopsies can be avoided in these patients and early detection can be achieved. These results further underscore the capability of the proposed XGB-AS-1 model to discern subtle differences between AS and non-AS candidates, thereby aiding in identifying AS candidates based on MRI.

Overall, 13 radiomics features that exhibited significant differences in feature values between AS and non-AS candidates were included in XGB-AS-1. Among them, the original _shape_ sphericity was greater for AS than for non-AS. This feature measures the similarity between the shapes of a lesion and a sphere. Thus, our findings suggest that lesions in AS candidates exhibit a more regular shape than those in non-AS candidates. Similar to our findings, Wang et al. [31] reported that the original _shape_ sphericity of adrenal lipid-poor adenomas was greater than that of adrenal metastases. This original_ shape_ sphericity was calculated from the original T2W images rather than from their derived images (e.g., wavelet images). Thus, differences in the original_ shape_ sphericity can provide radiologists and urologists with direct visual and semantic information to determine AS. Additionally, the selected features included wavelet and LBP-3D features, consistent with recent radiomics studies that reported a relationship between these features and tumor progression, as observed in Hodgkin lymphoma [32], cervical cancer [33], and meningioma [34]. Unlike original_ shape_ sphericity, these features are quantified from the derived images and, hence, are not visually represented. However, they comprise most of the selected radiomics features and encompass substantial subtle and invisible information capable of quantitatively characterizing the heterogeneity of PCa. Consequently, they play an important role in the identification of AS candidates.

This study had three limitations. First, although the proposed model was tested using the EVC, the study was retrospective. Future studies should validate and broaden our findings by using prospective data. Second, multicenter cases were manually segmented, which was time-consuming. An automatic segmentation algorithm would be beneficial for future studies. Third, the performance of XGB-AS-1 in ambiguous and intermediate-risk cases is not excellent; perhaps, a more advanced model (i.e., a deep-learning model) has the potential to stratify them accurately. However, the number of ambiguous and intermediate-risk cases in the current dataset was relatively small, rendering it insufficient to train and validate a deep-learning model. Further studies should develop more advanced models with a large amount of data, owing to the clinical significance of the risk re-stratification of ambiguous and intermediate-risk patients.

## Conclusions

In conclusion, the proposed radiomics model demonstrated promising performance in identifying candidates for AS, particularly in the classification of AS and non-AS candidates among the patients with PCa considered intermediate risk and those misclassified by the PI-RADS assessment. These findings suggest that the XGB-AS model has the potential to help identify patients who are suitable for AS and allow non-invasive monitoring of patients with AS, thereby reducing the number of annual biopsies and the associated risks of bleeding and infection.

### Supplementary Information

Supplementary Material 1.

## Data Availability

The clinical data used in this study are not available because they contain personal privacy information of patients unless the institutional approvals are obtained and the agreements of data usage are signed. However, the other materials of this study are available through a reasonable request to the corresponding authors.

## Abbreviations

ACC: Accuracy
AS: Active surveillance
AUC: Area under the receiver operating characteristic curve
csPCa: Clinically significant prostate cancer
EVC: External validation cohort
FIR: Favorable intermediate-risk
GGG: Gleason grade group
H: Hospital
LBP-3D: Local binary pattern in 3D
MRI: Magnetic resonance imaging
NCCN: National Comprehensive Cancer Network
PCa: Prostate cancer
PI-RADS: Prostate Imaging-Reporting and Data System
PSA: Prostate-specific antigen
ROI: Region of interest
RP: Radical prostatectomy
SEN: Sensitivity
SPE: Specificity
T2W: T2-weighted
TC: Training cohort
TRUS: Transrectal ultrasound
UFIR: Unfavorable intermediate-risk
XGB-AS: The radiomics model based on the eXtreme Gradient Boosting architecture for identifying active surveillance candidates
XGBoost: EXtreme Gradient Boosting
DT: Decision tree
LR: Logistic regression
RF: Random forest
AdaBoost: Adaptive boosting
IQR: Interquartile range
cT: stage clinical tumor stage
DCE: Dynamic contrast-enhanced
DWI: Diffusion-weighted imaging
mpMRI: Multiparametric magnetic resonance imaging
IR PCa: Immediate-risk prostate cancer
LASSO: Least absolute shrinkage and selection operator
MR: Magnetic resonance
