# Supplementary material for: Non-invasively identifying candidates of active surveillance for prostate cancer using magnetic resonance imaging radiomics
Source: Vis Comput Ind Biomed Art. 2024 Jul 5;7:16. doi: 10.1186/s42492-024-00167-6 (PMC11226574; doi:10.1186/s42492-024-00167-6)
Supplement: Supplementary file 1 — Supplementary Material 1. [file 42492_2024_167_MOESM1_ESM.docx]

**Supplementary material**

**Supplementary Section 1: Prostate Imaging Reporting and Data System (PI-RADS) assessment and lesion annotation**

The magnetic resonance (MR) images of prostates from hospital (H) 1, H2, H4, and H6 were divided into five groups and assessed by five junior radiologists (JR1–5). Subsequently, the same images were divided again into two groups, and their PI-RADS score results were verified by two expert radiologists (ER1 and ER2). Images from H3 and H5 were assessed by one (JR6) and two (JR7 and JR8) junior radiologists, respectively, and the results were verified by the third expert radiologist (ER3).

The prostate lesions were delineated on the T2-weighted (T2W) images by the same junior radiologists who had performed the PI-RADS assessment [1] on the corresponding lesions (i.e. JR1–8). The delineation of the prostate lesions was performed twice in a slice-by-slice manner by the same junior radiologist. The delineated region was referred to as the region of interest (ROI), based on which the subsequent radiomics feature would be extracted. The ITK-snap open-source software (v. 3.80) was used to delineate the lesions.

**Supplementary Section 2: Image pre-processing**

The magnetic resonance imaging (MRI) images of all patients were exported in the Digital Imaging and Communications in Medicine (DICOM) format through the post-processing workstation. Subsequently, the DICOM-format images were converted into the Neuroimaging Informatics Technology Initiative format, removing the patients’ personal information, such as name, birth date, and age at the time of examination. Differences in acquisition parameters and MRI scanner manufacturers can result in inter-center differences in image intensity and image spatial resolution. To reduce inter-center variability, an image harmonization method was used. First, each original T2W image was normalized by subtracting the mean and then being divided by the standard deviation. Then, the normalized image was resampled to achieve a uniform pixel spacing of 1 × 1 × 1 mm^3^.

**Supplementary Section 3: Extraction of radiomics features**

Radiomics features were extracted from T2W images and their derived images using the open-source package of Pyradiomics (v.3.0.1, https://pyradiomics.readthedocs.io/en/v3.0.1/) in Python (v.3.7, https://www.python.org/). A total of 1,595 radiomics features were extracted from the ROI of each patient. Specifically, 107 radiomics features were extracted from the ROI of each original T2W image, including the following seven types: 18 first-order statistics (first-order) features, 14 shape-based features, 24-grey level co-occurrence matrix (GLCM) features, 16 grey-level run length matrix (GLRLM) features, 16 grey-level size zone matrix (GLSZM) features, 5 neighboring grey-tone difference matrix (NGTDM) features, and 14 grey-level dependence matrix (GLDM) features. Additionally, all radiomics features mentioned above, except the shape-based features (i.e. first-order, GLSZM, GLCM, GLRLM, NGTDM, and GLDM), were also extracted from the ROI in seven types of derived images, obtained by applying seven different filters to the original T2W image, namely, gradient, wavelet, logarithm, square, square root, exponential, and local binary pattern in 3D (LBP-3D). Among them, the wavelet filter was applied to the original T2W image to obtain 744 wavelet features. Specifically, for the wavelet transformation, high- (H) and low-pass (L) filters were applied in three dimensions (X, Y, and Z axes) to produce eight decomposition images, which were referred to as LLL, LLH, LHL, LHH, HLL, HLH, HHL, and HHH. From each of the eight decomposition images, six types (first-order, GLSZM, GLCM, GLRLM, NGTDM, and GLDM) of radiomics features were extracted. Further, for the derived images obtained using filters such as gradient, logarithm, square, square root, and exponential filters, 465 radiomics features were extracted. Finally, for the derived images obtained using LBP-3D filters with lbp3D levels of 2, lbp3DIcosphereRadius of 1, and lbp3DIcosphereSubdivision of 1, 279 radiomics features were extracted. Thus, for each original T2W image and its derived images, 1,595 radiomics features were extracted.

**Supplementary Section 4: Feature selection**

Because a three-fold cross-center validation was used to develop and externally validate the radiomics models in the current study, feature selection was performed in the training cohort for each fold of cross-center validation. Specifically, for each fold of the cross-center validation, all 1,595 radiomics features extracted from the T2W images and their derived images of the training cohort were standardized as Z-scores by subtracting their respective means and then dividing the result by their respective standard deviations. The radiomics features from the external validation cohort were standardized using the same mean and standard deviation as those for the training cohort. Feature selection based on a two-sample Student’s t-test was performed. The features with significant differences between the active surveillance (AS) and non-AS groups were retained. The least absolute shrinkage and selection operator (LASSO) regression was then adopted for further feature selection. The parameter alpha within LASSO was set using a five-fold cross-validation, which is a commonly used method of adjusting parameters. LASSO with the optimal alpha was then performed in the training cohort. The features with non-zero coefficients were selected to develop the radiomics model.

**Supplementary Section 5: Definition of intermediate-risk cases**

According to the National Comprehensive Cancer Network guidelines [2], a patient with prostate cancer (PCa) is classified as having intermediate risk if they have no high-risk or very high-risk group features, and have one or more intermediate-risk factors (IRFs; i.e. cT2b–cT2c, Gleason grade group [GGG] 2 or 3, and prostate-specific antigen level 10–20 ng/mL). Patients with intermediate-risk PCa are classified as having favorable intermediate-risk (FIR) and unfavorable intermediate-risk (UFIR). A patient classified as having FIR is characterized as an individual with PCa who exhibits all of the following factors: one IRF, GGG 1 or 2, and <50% positive biopsy cores (PBCs). In contrast, a patient with UFIR refers to an individual with PCa who has one or more of the following factors: two or three IRFs, GGG 3, and ≥50% PBCs.

**Supplementary Table S1** Magnetic resonance imaging sequence parameters

|  | Hospital 1  (*n*=166) | Hospital 2  (*n*=167) | Hospital 3  (*n*=97) | Hospital 4  (*n*=100) | Hospital 5  (*n*=316) | Hospital 6  (*n*=110) |
| --- | --- | --- | --- | --- | --- | --- |
| **MRI strength and vendor** | 3.0 T,  Siemens Skyra | 3.0 T,  Philips Ingenia | 3.0 T,  Siemens Skyra | 3.0 T,  Philips Achieva TX | 3.0 T,  Siemens,  GE, and Philips | 3.0 T,  Siemens Skyra and Vero |
| **B value (s/mm^2^)** | 50, 70, 1500, 2000 | 10, 20, 50, 100, 200, 1000, 2000 | 0, 1500 | 0, 1000, 2000 | 0, 1000, 2500 | 0, 50, 800, 1000, 1500 |
| **Slice thickness (mm)** | 3 | 1.5, 2, 3, 3.4, 3.5, 3.7, 3.8, 3.9, 4, 4.1, 4.2, 4.3, 5 | 3, 5 | 3 | 3, 3, 3 | 3.5, 4, 5, 5.5 |
| **Spacing between**  **slices (mm)** | 3, 3.45 | 1.65, 3, 3.2, 3.3, 3.4, 3.5, 3.7, 3.8, 3.9, 2.2, 4, 4.1, 4.2, 4.3, 5 | 0 | 3 | 0 | 3.5, 4, 4.8, 6, 6.6 |
| **Echo time (s)** | 60, 104 | 77, 78, 100 | 13, 78, 104 | 76, 80 | 126, 100, 110 | 62, 64, 74, 97, 104 |
| **Repetition time (s)** | 6540, 7590 | 4542, 4828, 4898, 4733, 4972, 6000 | 807, 4300, 6500, 6880 | 2750, 3000 | 6000, 4652, 3424 | 4480, 5000, 5100, 7500, 8600 |

Abbreviations: *T2WI,* T2-weighted imaging; *DWI,* diffusion-weighted imaging; *ADC,* apparent diffusion coefficient; *MRI*, magnetic resonance imaging

**Supplementary Table S2** Details in the data splitting for each fold of the three-fold cross-center validation

| No. of 3-fold cross-center validation | TC | EVC | Threshold |
| --- | --- | --- | --- |
| Fold 1 | H1, H2, H3, H4  (n=530, AS=91, AS%=17.3%) | H5, H6  (n=426, AS=55, AS%=12.9%) | 0.172  (91/530) |
| Fold 2 | H1, H2, H5, H6  (n=759, AS=121, AS%=15.9%) | H3, H4  (n=197, AS=25, AS%=12.7%) | 0.159  (121/759) |
| Fold 3 | H3, H4, H5, H6  (n=623, AS=80, AS%=12.8%) | H1, H2  (n=333, AS=66, AS%=19.8%) | 0.128  (80/623) |

Note: The classification threshold was determined by threshold-moving method, namely,$\frac{n_{AS}}{n_{AS}+n_{non-AS}}$, where $n_{AS}$ and $n_{non-AS}$ refer to the number of AS and non-AS cases in TC, respectively.

Abbreviations: *TC,* training cohort; *EVC,* external validation cohort; *AS%,* the proportion of active surveillance candidates

**Supplementary Table S3** Optimal hyperparameters of three XGB-AS models generated by three-fold cross-center validation, respectively

| Models | Parameters |
| --- | --- |
| XGB-AS-1 | learning_rate=0.01,  n_estimators=480,  max_depth=6,  min_child_weight=1,  gamma=0.1,  subsample=0.8,  reg_lambda=10,  colsample_bytree=0.5,  nthread=4,  scale_pos_weight=3,  seed=6,  objective='binary:logistic',  reg_alpha=0.0 |
| XGB-AS-2 | learning_rate=0.01,  n_estimators=400,  max_depth=5,  min_child_weight=1,  gamma=0.1,  subsample=0.6,  reg_lambda=10,  colsample_bytree=0.5,  nthread=4,  scale_pos_weight=3,  seed=8,  objective='binary:logistic',  reg_alpha=0.2 |
| XGB-AS-3 | learning_rate=0.1,  n_estimators=50,  max_depth=2,  min_child_weight=1,  gamma=0.1,  subsample=0.6,  reg_lambda=10,  colsample_bytree=0.5,  nthread=4,  scale_pos_weight=3,  seed=4,  objective='binary:logistic',  reg_alpha=0.1 |

Abbreviations: *AS,* active surveillance; *XGB-AS-1,* the XGB-AS model developed in the first fold of the three-fold cross-center validation; *XGB-AS-2,* XGB-AS model developed in the second fold of the three-fold cross-center validation; *XGB-AS-3*, XGB-AS model developed in the third fold of the three-fold cross-center validation.

**Supplementary Figure S1** Thirteen radiomic features selected by the feature selection process for the development of the XGB-AS-1 and their respective coefficients


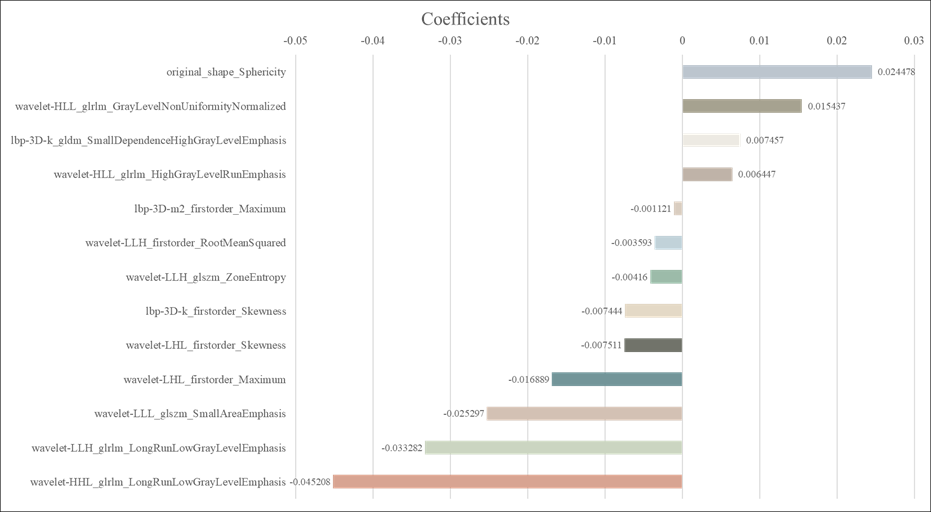


Abbreviations: *XGB-AS-1,* the most clinically applicable radiomics model developed in the current study based on the eXtreme Gradient Boosting architecture for identifying active surveillance candidates

**References**

1. Turkbey B, Rosenkrantz AB, Haider MA, Padhani AR, Villeirs G, Macura KJ et al (2019) Prostate imaging reporting and data system version 2.1:2019 update of prostate imaging reporting and data system version 2. Eur Urol 76(3):340-351. https://doi.org/10.1016/j.eururo.2019.02.033

2. National Comprehensive Cancer Network (NCCN) clinical practice guidelines in oncology: prostate cancer. version 3.2024 [cited May 10 2024]. Available from: https://www.nccn.org/guidelines/guidelines-detail?category=1&id=1459
